# Intermittent Hypoxia in Preterm Neonates and Its Effect on Neonatal Morbidity and Mortality: A Systematic Review

**DOI:** 10.7759/cureus.45561

**Published:** 2023-09-19

**Authors:** Vinod Kumar Mandala, Suresh Kumar Yadav Bollaboina, Baburao Changala, Rakesh Kotha, Lingareddy Kasula

**Affiliations:** 1 Pediatrics, Kakatiya Medical College, Warangal, IND; 2 Pediatrics, Niloufer Hospital, Hyderabad, IND; 3 Pediatric Surgery, Niloufer Hospital, Hyderabad, IND; 4 Neonatology, Osmania Medical College, Hyderabad, IND; 5 Neonatology, Niloufer Hospital, Hyderabad, IND

**Keywords:** hypoxia, hypoxemia, neurodegenerative diseases, retinopathy of prematurity, bronchopulmonary dysplasia

## Abstract

The goal of the present systematic review was to investigate the occurrence patterns of intermittent hypoxemia in newborns throughout the early postnatal period as well as the link between neonatal intermittent hypoxemia exposure and harmful consequences such as neonatal morbidity and death. We collected data from 2014 to 2023 using several abstracting, referencing, and indexing database libraries in the field of medical sciences. A total of 715 papers were evaluated by both authors, and only seven articles met the specified review criteria after a thorough analysis. In preterm neonates with intermittent hypoxia (IH), severe morbidities such as bronchopulmonary dysplasia (BPD), retinopathy of prematurity (ROP), motor impairment, and cognitive delay were found. Only one study that extended to 18 months noted mortality. The length and occurrence of intermittent hypoxemia and the stage of premature neonates at the time of delivery are all closely associated with these morbidities. Therefore, it becomes important to continuously measure the patterns of occurrence of intermittent hypoxemia during early postnatal life to avoid its long-term morbidity and mortality impact.

## Introduction and background

Premature births, or preterm births, are those that occur before 37 weeks of pregnancy. Its rate is around 4% to 16% of all births across the countries [1]. Preterm labor is a major contributor to perinatal morbidity and death in both developed and developing nations, complicating 5% to 10% of pregnancies [2].

Poor outcomes and extended hospital stays are known to be impacted by the absence of stability in the respiratory system and associated oxygenation in preterm deliveries. Preterm newborns experience bouts of apnea and intermittent hypoxia (IH) in the early postnatal period because of poor respiratory control combined with a developing lung [3].

Intermittent hypoxia is often referred to as episodic hypoxia, which involves alternating periods of normoxia and hypoxia. Hypoxia is any oxygen level below that of normoxia. An IH event is commonly defined in clinical research studies as a decrease in oxygen saturation to at least 80%. In contrast, pulse oximeter hypoxemia alarms in neonatal intensive care units are typically set at 85% and 90% to meet the American Academy of Pediatrics guidelines for oxygen saturation of 90% to 95% [3]. Normoxia is defined as exposure to oxygen levels typically present in the earth's atmosphere (21% O2) [4]. The main causes of IH episodes include the consequences of immature respiratory control, which causes apnea and breathing pauses, as well as inadequate and/or blocked inspiratory attempts. However, limited lung capacity and low pulmonary oxygen storage obviously predispose respiratory pauses to quickly cause a desaturation event. In babies with bronchopulmonary dysplasia (BPD), this chain of events is amplified. The respiratory morbidities linked to IH episodes may thus be expected. Intermittent hypoxia episodes have been linked to a number of long-term morbidities other than respiratory, including retinopathy of prematurity (ROP), wheezing, BPD, and neurodevelopmental damage [5].

Martin et al. noted that instances of intermittent hypoxia gradually rise in extremely low-birth-weight newborns throughout the first four weeks of postnatal life, reaching a plateau at weeks four to six and then slowly declining from weeks six to eight [6]. Intermittent hypoxia is related to intermittent hypoxemia events, which are common during early postnatal life, particularly in preterm infants. These events have also been linked with multiple morbidities caused by IH. The patterns (frequency, length, and timing) of IH episodes do play a role in the link between IH and unfavorable consequences [7].

Additionally, a variety of variables put preterm neonates at high risk for developing intermittent hypoxemia. Some of these factors include respiratory immaturity, anemia, and lung disease. Brief bouts of oxygen desaturation may have a cumulative effect on neonatal health despite their apparent clinical insignificance [8]. The present systematic review focuses on neonatal morbidity and mortality patterns in preterm infants suffering from IH.

## Review

Method

Search Strategy

The literature on IH in neonates and its association with multiple morbidities and mortality was thoroughly evaluated. Data was collected using several abstracting, referencing, and indexing databases in the field of medical sciences, such as Embase, PubMed, Medline, CINAHL, Wiley, Science Direct, Web of Science, and Google Scholar. Keywords such as morbidity, mortality, cognition, memory, retinopathy, wheezing, bronchopulmonary dysplasia, and sleep apnea were utilized in various combinations with IH in infants. Additionally, manual searches were done by looking through the reference lists of the studies that were included. We focused on data available between 2014 and 2023 for the present study. We derived medical subject headings (MeSH) using thematic keywords. We used the boolean operators "OR" and AND, parentheses (), and field codes. We searched both the text and abstracts.

The search strategy was initially developed for MEDLINE and modified for other databases. We searched the literature using the phrases "intermittent hypoxia in infants", "mortality due to intermittent hypoxia in infants", and "retinopathy due to intermittent hypoxia in infants". The keywords used were "intermittent hypoxia", "intermittent hypoxemia", "neonates", "newborns", "bronchopulmonary dysplasia", "retinopathy of prematurity", "mortality", and "morbidity". These keywords are used in combination with the boolean operators "OR" and AND, parentheses (), and field codes as mentioned above. Before starting the investigation, we established a well-described protocol.

Identification of the Study

All citations were collected and exported to EndNote (Clarivate, London, UK). The duplicates collected through various search engines were eliminated. All citations were screened through titles, abstracts, and the full text of all appropriate research. A total of 800 potential literature citations were found. Two of the reviewers looked over their titles and abstracts to see if they should be included. Submissions from additional sources, such as personal files and a study of citations in the bibliographies of included studies, were also accepted by participating reviewers. Discrepancies were revealed with the third reviewer to attain harmony.

Data Extraction

The following were considered inclusion criteria for the present study: evidence from at least one adequately constructed randomized controlled trial (RCT); evidence from carefully planned non-randomized trials; data from cohort or case-control studies; fully accessible texts published in peer-reviewed journals; and studies focusing directly on IH in preterm neonates and its effect on neonatal morbidity and mortality. Exclusion criteria included conference articles with only abstracts, editorial comments, guidelines, policies, or treatment recommendations, and studies focusing only on patients older than two years.

Identification of Included Studies

**Figure 1:**
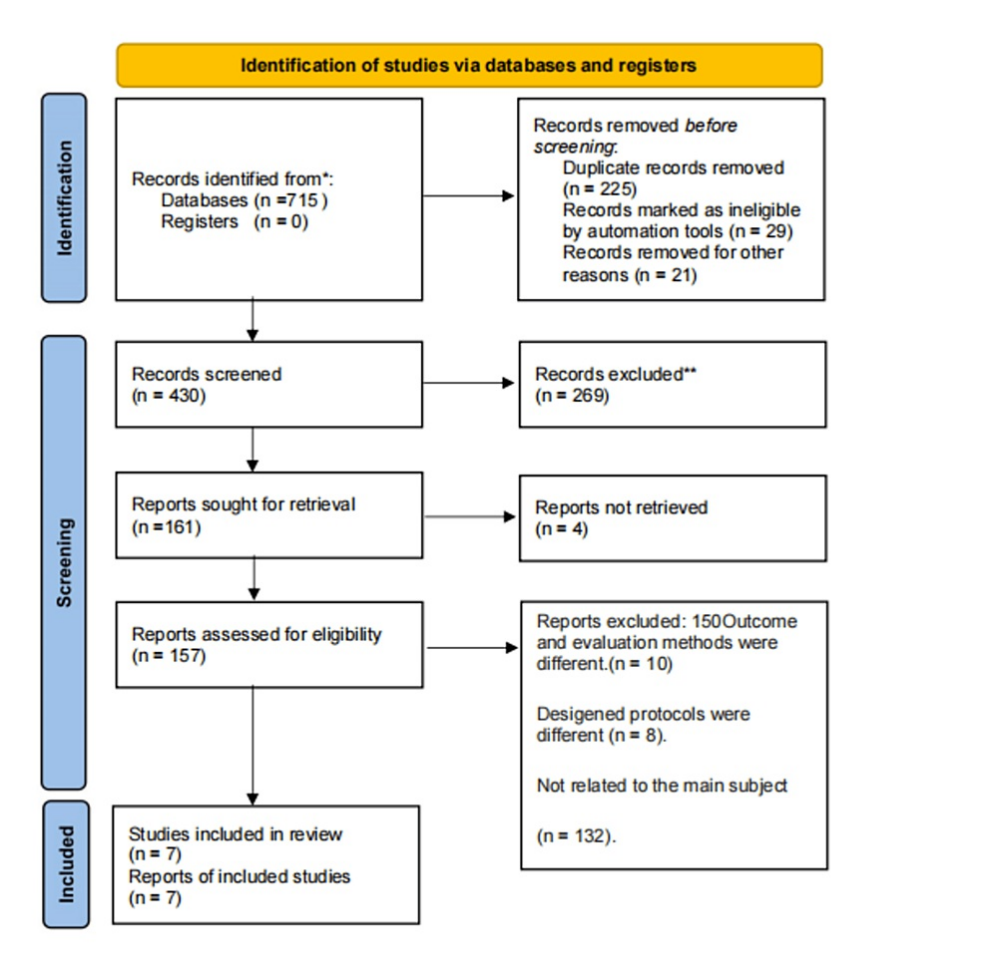
PRISMA flow diagram PRISMA: Preferred Reporting Items for Systematic Reviews and Meta-Analyses

The initial search resulted in 715 potentially relevant research studies. After reviewing the titles, it was discovered that 275 research papers were duplicates, so the abstracts of 430 articles were reviewed. Among them, 269 did not fit the inclusion criteria. Further full text was evaluated for 161 papers, and only six papers were discovered after a thorough analysis of the whole text to meet the specified review criteria. The criteria were intermittent hypoxia in human neonates after preterm delivery. One more eligible study was identified after reference lists from eligible studies were reviewed. Based on a careful examination of full-text data, only seven studies from different parts of the world were included (Figure 1).

Overall, four (57.1%) of the seven articles reviewed demonstrated any of the adverse effects of hypoxia. Out of them, only one case study (14.3%) reported mortality with morbidity. Three studies only measured the frequency of hypoxia in preterm neonates after birth.

Quality Assessment

To assess the quality of studies included in the present systematic review, the revised assessment of multiple systematic reviews (R-AMSTAR) was followed. The Newcastle-Ottawa scale was chosen as the appropriate risk of bias tool. This tool contains three sections, namely: (1) selection, with four questions and a maximum of one star given for each numbered item; (2) comparability, with a maximum of two stars given to each study; and (3) outcome, which has four questions containing a single star. The total value on this scale is 10, which includes a series of nine questions containing 10 points.

The quality of the systematic review paper was assessed using the Newcastle-Ottawa scale, which has a total of 9 points. Four studies got 9 points, and three studies got 8 points. It shows that the papers included in the present study are of good quality (Table 1).

**Table 1:** Result of Newcastle-Ottawa scale on systematic review * indicates no.of points scored

N	Study	Selection	Comparability	Outcome
1.	Poets et al., 2015 [9]	***	**	****
2	Jenson et al., 2021 [10]	***	**	****
3	Raffay et al., 2019 [11]	***	**	****
4	Williams et al., 2019 [12]	***	**	***
5	Fiore and Raffay, 2021 [7]	***	**	***
6	Wellington et al., 2019 [13]	***	**	***
7	Alalaiyan et al., 2023 [14]	***	**	****

Results

We included a total of seven studies after screening them in our systematic review. The included studies were of very good quality, which we assessed objectively through the Newcastle-Ottawa scale. Three studies got 8 points, and four studies got 9 points. Our studies were published between 2015 and 2023, and as such, IH has only recently been observed. All trials were observational studies. Most of the studies included extremely preterm babies. Only one study included late preterm babies. Intermittent hypoxia is usually more common in very preterm neonates. Only one study monitored up to 18 months; most of the studies followed up to 36 weeks and 45 weeks. Outcome as a measure of mortality was included in only one study. The outcomes of the studies were BPD in two cases, ROP in two cases, and mortality and neurodevelopment in one case (Table 2).

**Table 2:** Summary of the evidence of intermittent hypoxia in preterm neonates BPD: Bronchopulmonary dysplasia

N.	Authors	Gestational age	Period of monitoring	Morbidity	Mortality
1	Poets et al., 2015 [9]	23 weeks 0 days through 27 weeks 6 days	18 months after birth	Severe retinopathy of prematurity motor impairment, cognitive delay	Died or survived with disability
2	Jenson et al., 2021 [10]	23 weeks 0 days –27 weeks 6 days	Until at least 36 weeks	Severe BPD	-
3	Raffay et al., 2019 [11]	(24 weeks 0 days –27 weeks 6 days	36 weeks	BPD	-
4	Williams et al., 2019 [12]	34 weeks 0 days –36 weeks 6 days	45 weeks	-	--
5	Fiore et al., 2019 [7]	Compared extremely (<28 weeks), very (28–<32 weeks) and moderately (32–<34 weeks) preterm infants	45 weeks	-	-
6	Wellington et al., 2019 [13]	Infants <32 weeks		-	-
7	Alalaiyan et al., 2023 [14]	24 weeks 0 days –36 week 6 days -	For 7 days	Retinopathy of prematurity	-

All studies noted that higher frequencies of IH events of longer durations and greater time with hypoxemia and very early and moderately preterm infants are more prone to morbidities and mortalities [11,12,7,13]. Poets et al. investigated the link between intermittent hypoxemia and late mortality or impairment in very preterm children [9]. In 25 hospitals across six nations, they gathered information from the Canadian Oxygen Trial. Ages 23 weeks, 0 days to 27 weeks, and preterm 6 days were contemplated. They noted that a higher number of intermittent hypoxemic events lasting at least a minute was linked to an increased risk of late mortality or disability at 18 months. Following persistent hypoxemia, relative risks for all secondary outcomes rose in a similar manner. Bradycardia had little impact on how well hypoxemia predicted outcomes. They noted severe ROP, motor impairment, and cognitive delay [8]. Recently, another study by Alalayian et al. performed overnight pulse oximetry on 50 preterm newborns with a birth weight of 1500 grams or less. Among them, half (50%) had mild hypoxia, 28% had severe hypoxia, 20% had moderate hypoxia, and 2% had no hypoxia, according to the McGill score. Desaturations were more common (62.5%) in newborns who weighed no more than 1000 grams. They noted retinopathy of prematurity (ROP) stages 1 and 2 in half of the babies, whereas stage 3 was in 14% and stage 4 in 2%. They claimed that 8% of babies needed surgical intervention for ROP [14].

Bronchopulmonary dysplasia was observed at 36 weeks postmenstrual age among early preterm neonates (24 to 27 weeks gestation) in two studies independently conducted by Jensen et al. and Raffay et al. [10,11]. They also noted that the length of exposure and the number of hypoxemic episodes increase the relative risk of severe BPD. Significant disparities in the frequencies of hypoxemia between newborns with and without severe BPD developed within the first week after delivery, according to Raffay et al. (2019). They also reported that severe BPD occurred in 32.6% of cases [11].

Discussion

The purpose of this systematic review was to identify the adverse effects of IH on preterm neonates. This is the first of this kind of review that summarizes BPD, ROP, and neurodevelopmental abnormalities such as motor impairment and cognitive delay. Only one study continued until 18 months and noted the deaths of neonates [9]. Further long-term collaborative studies are required to determine the impact of IH on the mortality of preterm neonates. This review noted that BPD, ROP, neurodevelopmental abnormalities such as motor impairment, and cognitive delay were noted as major morbidities in preterm neonates with intermittent hypoxia [9,10,14].

Before the mid-1970s, intermittent arterial sampling formed the basis for the assessment and management of supplemental oxygen administration. There was no clear evidence of frequent fluctuations in preterm infants. In 1990, after the invention of pulse oximetry, it became more evident in preterm infants.

Inadequate respiratory control, leading to apneas and pauses in breathing, and ineffective and/or obstructed inspiratory efforts are the main triggers for IH events. However, several physiological parameters likely contribute to the resulting desaturation, particularly low lung volumes that are exacerbated by hypoxia episodes in intrauterine growth restriction (IUGR) infants. Preterm infants with IH, although ventilated due to inadequate respiratory support and low lung volumes, will develop hypoxia episodes. As per the literature, hypoxemic episodes occur during the first week of postnatal life, with a progressive increase in weeks two to three, a plateau around weeks four to six, and finally a decrease in weeks six to eight, usually consistent with our study [15].

Optimization of baseline oxygenation with oxygen and continuous positive airway pressure (CPAP) is the primary treatment. Xanthine therapy, particularly caffeine, has become a mainstay of neonatal care, improving not only respiratory but also neurodevelopmental outcomes. Another study documented a decrease in apnea associated with desaturation and bradycardia in the 12 hours following a red blood cell transfusion. Hypoxia intensity and duration were the main predictors [8].

In some animal trials, brief hypoxemia is beneficial, and less than one minute of IH is not associated with any adverse outcome. Intermittent hypoxia is used as a treatment in some adult cases of sleep apnea and hypertension. However, our review states that IH is very harmful to babies [16-18].

## Conclusions

With increasing preterm births and advances in medical management, many extreme preterm babies survive, which is why we are seeing many cases of IH. As per our systematic review, BPD, ROP, and neurodevelopmental abnormalities such as motor impairment and cognitive delay were noted as major morbidities in preterm neonates with IH. These morbidities are highly correlated with the duration and occurrence of intermittent hypoxemia and the stage of preterm neonates. These babies need long-term follow-up. Some small episodes will not be recorded in pulse oximetry; for them, documentation and follow-up are needed for further research. As per present knowledge, to avoid IH episodes, short-term oxygen supply and the implementation of fraction of inspired oxygen (FiO2) automated controllers are needed. New research regarding the cause and management of IH is required for not just survival but intact survival.
